# Data on the cultivation of *Prochloron* sp. at different salinity levels

**DOI:** 10.1016/j.dib.2020.105241

**Published:** 2020-02-01

**Authors:** Inneke Fenny Melke Rumengan, Triana Mansye Kubelaborbir, Trina Ekawati Tallei

**Affiliations:** a Department of Marine Science, Faculty of Fisheries and Marine Sciences, Sam Ratulangi University, Manado, Indonesia; b Department of Aquatic Resources Management, Faculty of Agriculture, Forestry, and Marine, University of Ottow Geissler, Jayapura, Indonesia; c Department of Biology, Faculty of Mathematics and Natural Sciences, Sam Ratulangi University, Manado, Indonesia

**Keywords:** *Prochloron*, Cultivation, Salinity, Ascidian

## Abstract

The data in this article describe the population growth of *Prochloron* cells outside the hosts at the different salinity levels. The cultivation was performed in enriched standard culture media with continuous photoperiod. The culture stock of *Prochloron* cells which was made as inoculum (starter) in the laboratory was isolated from tunics of an ascidian *Lissoclinum patella*. The ascidian was obtained from 20 m depth at Malalayang coastal water in Manado Bay, North Sulawesi, Indonesia. The initial stock was kept in 20 ppt liquid medium. Then the cells were transferred into culture chambers, each prepared with different salinities: 15, 20, 25, 30, 35, 40 and 45 ppt. After the cells reached exponential phase, some drops of cell suspension were transferred into agar media with the same salinity level until the green colony appeared. Each of the colonies was transferred again into liquid media with the same salinity. Population growth was observed until the death phase. The results of the study show that: (1) the growth of *Prochloron* cells at different salinity showed a different growth rate; (2) *Prochloron* cells grew well in salinity of 15 and 35 ppt; (3) the maximum population growth of *Prochloron* from each treatment varied. *Prochloron* cells grown in a medium with 35 ppt salinity had a rapid adaptability to the new culture environment. However, the maximum population growth was reached on the 75th day with a cell density of 31.00 × 10^6^ cells/ml in a medium with 15 ppt salinity, much higher than those of the other treatments (20, 25, 30, 35 and 45 ppt). The data presented here are the success of the cultivation of *Prochloron* cells outside the host.

**Table:** Specifications Table

Subject area	*Aquatic Science*
More specific subject area	*Marine Biotechnology*
Type of data	*Table, text file, graph, figure*
How data was acquired	*Direct observation under microscope*
Data format	*Graphs*
Experimental factors	*Various salinity levels*
Experimental features	*Laboratory condition using natural seawater under continuous photoperiod*
Data source location	*Laboratory of Molecular Biology and Marine Pharmaceutics, Faculty of Fisheries and Marine Science, Sam Ratulangi University, Manado, Indonesia*
Data accessibility	*Data are included in this article* *The raw dataset is available from Mendeley data repository: DOI*:https://doi.org/10.17632/sh6bngkjtw.1

**Table:** 

**Value of the Data** • This is the first report on in vitro cultivation of *Prochloron* sp.; • The data give insight on how to get sustainable biomass of the *Prochloron* cells for further extracting the biological active compounds such as potent anticancer cyclic peptides. • The data can be regarded as a base line to develop a mass cultivation of the *Prochloron* as producer of the valuable biologically active compounds. The cultivated cells could be further studies by genomic approach to characterise many potential cluster genes encoding proteins of interest. The cultivated cells may produce novel compounds by gene cluster engineering.

## 1 Data

**Fig. 1:**
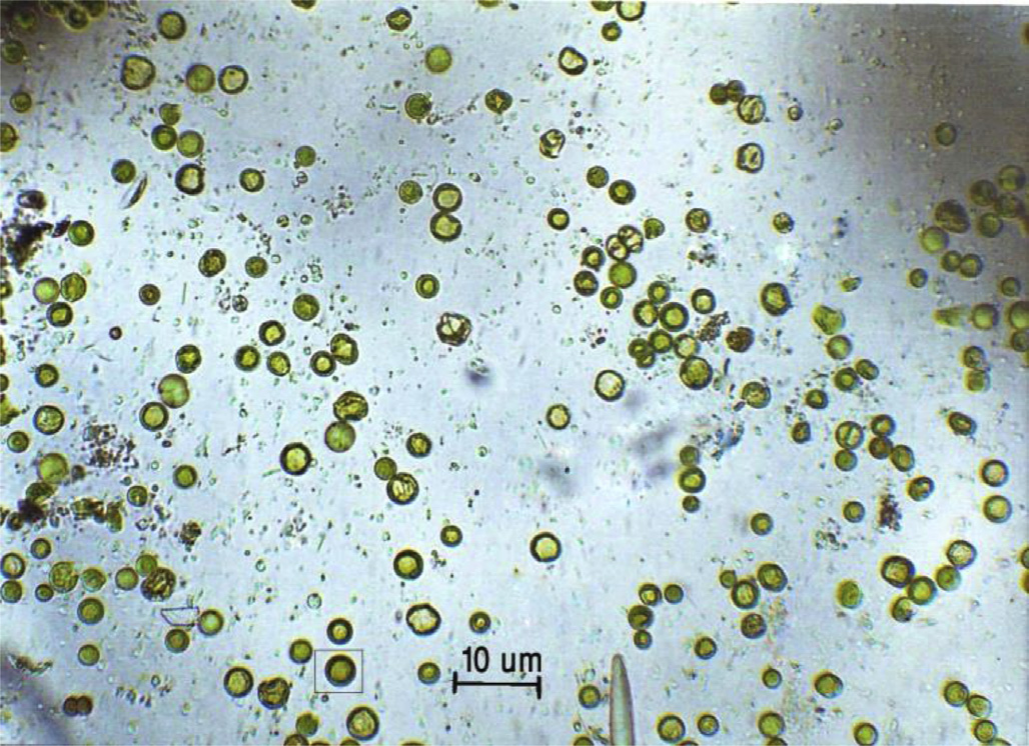
*Prochloron* cells in 20 ppt salinity enriched with the Hirata - medium in liquid culture stock as inoculum.

*Prochloron* is an obligate marine photosynthetic prokaryote found associated with didemnid ascidians [1]. This unicellular microbe has been reported to produce many biological active compounds including potential anticancer cyclic peptides [2,3]. The raw dataset of this experiment is available from https://doi.org/10.17632/sh6bngkjtw.1. In this experiment, *Prochloron* cells were grown on agar medium with 20 ppt salinity enriched with the Hirata medium to be used as a stock culture to be used further. The cells were green, round, single-celled and had a core area around the cell wall (Fig. 1.). However, their size was smaller than the original (wild type) cells obtained from its natural habitat. The time needed to grow *Prochloron* in the agar media was approximately one month. *Prochloron* growth began to appear on the 28th day, characterized by the growth of several colonies on the edge of the dish which was directly exposed to light. The growth lasted until the 32nd day.

**Fig. 2:**
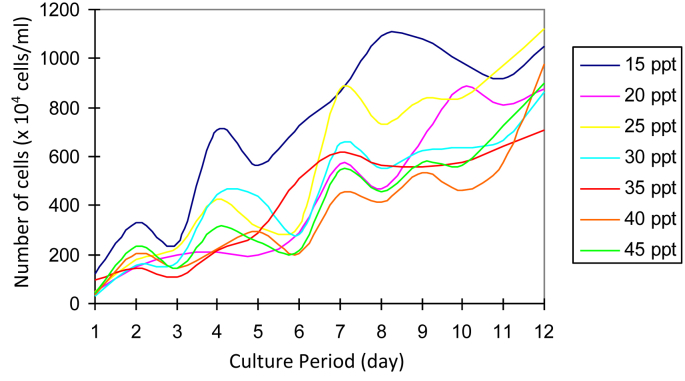
*Prochloron* cells growth after being transferred from liquid culture stock at 20 ppt salinity to newly prepared liquid media with different salinities.

**Fig. 3:**
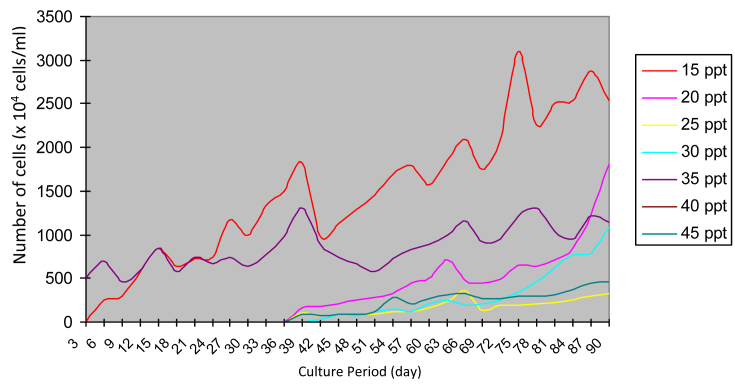
*Prochloron* cells growth after being transferred from solid media to liquid media with different salinity.

The growing colonies were then transferred into liquid media with various salinities. *Prochloron* cells showed slow growth at the beginning. The growth rate of the *Prochloron* population at different salinity varied starting from day 1 to day 12 (Fig. 2.). The addition of cell numbers was marked by the change in the color of the culture medium from the light green to dark solid green. From each liquid medium, the cells were then transferred to a solid medium with the same salinity. Subsequently, the growing colonies were transferred to newly prepared liquid medium with the same salinity. The growth profile of these cells is shown in Fig. 3. *Prochloron* cells grown in a medium with 35 ppt salinity had a rapid adaptability to the new culture environment. When transferred from the agar media into liquid media, 3 days later the cell had grown. The best growth occurred at 15 ppt salinity.

The maximum population growth of each treatment also varied. At 15 ppt salinity, the peak growth of the population occurred on day 75 with a cell density of 31.00 × 10^6^ cells/ml. At salinity of 20 ppt, the maximum population growth occurred on the 90th day with a cell density of 18.06 × 10^6^ cells/ml. Maximum population growth at 25 ppt salinity was observed on day 66 with cell density 3.55 × 10^6^ cells/ml. At 30 ppt salinity, the maximum population growth was reached on the 90th day with a cell density of 10.88 × 10^6^ cells/ml. Maximum population growth was obtained on day 78 at 35 ppt salinity with cell density 12.99 × 10^6^ cells/ml. Furthermore, in the 45 ppt salinity, the maximum population growth was achieved on day 87 with a cell density of 4.40 × 10^6^ cells/ml.

**Fig. 4:**
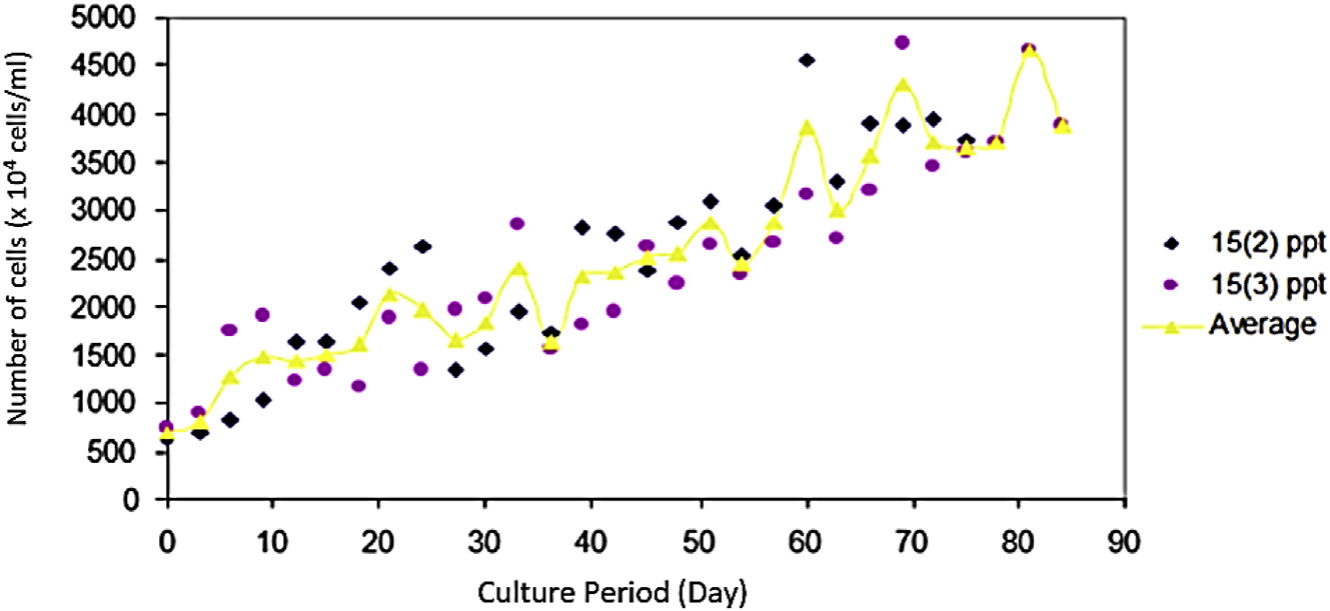
The absolute growth rate of the population of *Prochloron* cells at 15 ppt salinity.

**Fig. 5:**
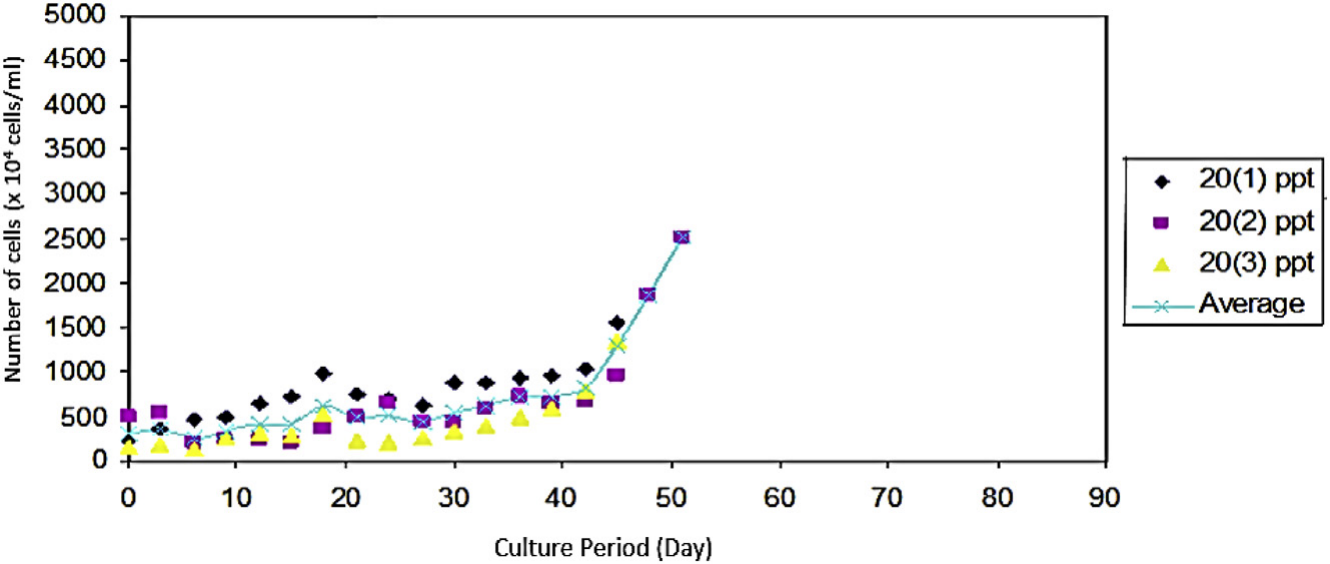
The absolute growth rate of the population of *Prochloron* cells at 20 ppt salinity.

Based on the total population value (Nt), the absolute population growth rate of *Prochloron* cells can be calculated. The absolute growth rate of the population of *Prochloron* cells at 15 ppt can be seen in Fig. 4. Acclimatization (lag phase) occurred at the beginning of growth, so that the absolute growth rate of the population was calculated when the cell began to grow. *Prochloron* cells cultured in medium with a salinity of 15 ppt had a population growth rate of 60.26 × 10^4^ cells/ml. Fig. 5 shows that *Prochloron* cells cultured in the medium with a salinity of 20 ppt had a population growth rate of 53.72 × 10^4^ cells/ml.

**Fig. 6:**
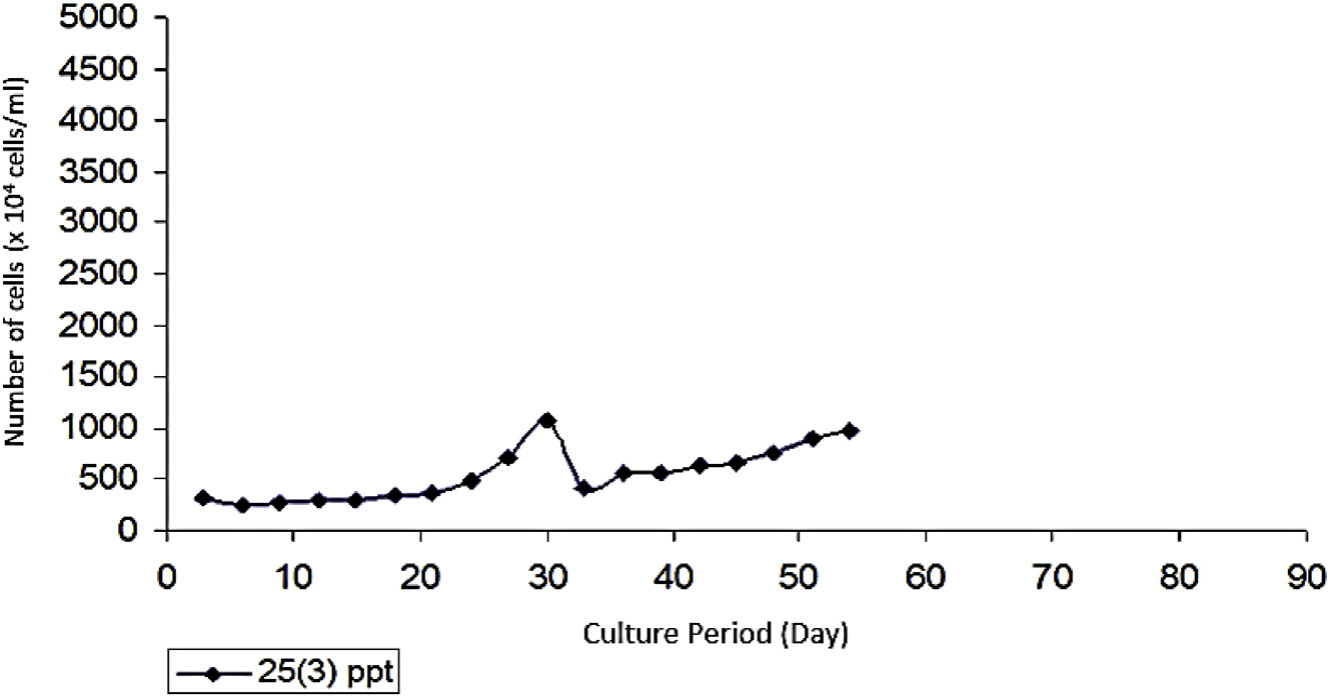
*Prochloron* cells growth at salinity of 25 ppt.

**Fig. 7:**
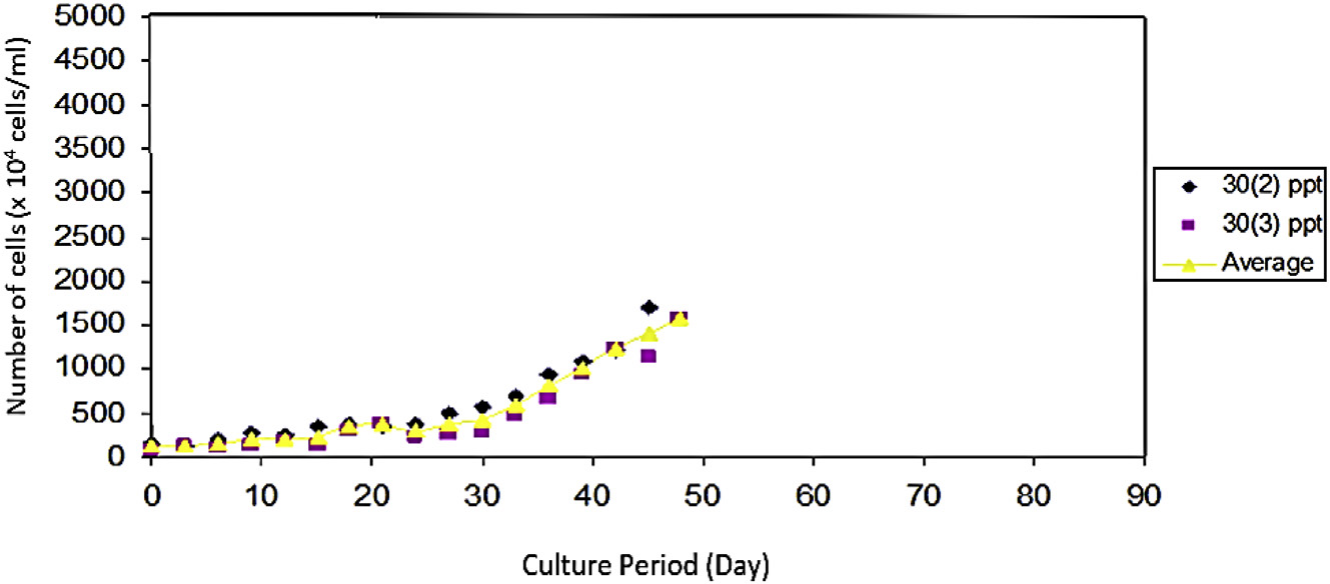
*Prochloron* cells growth at salinity of 30 ppt.

**Fig. 8:**
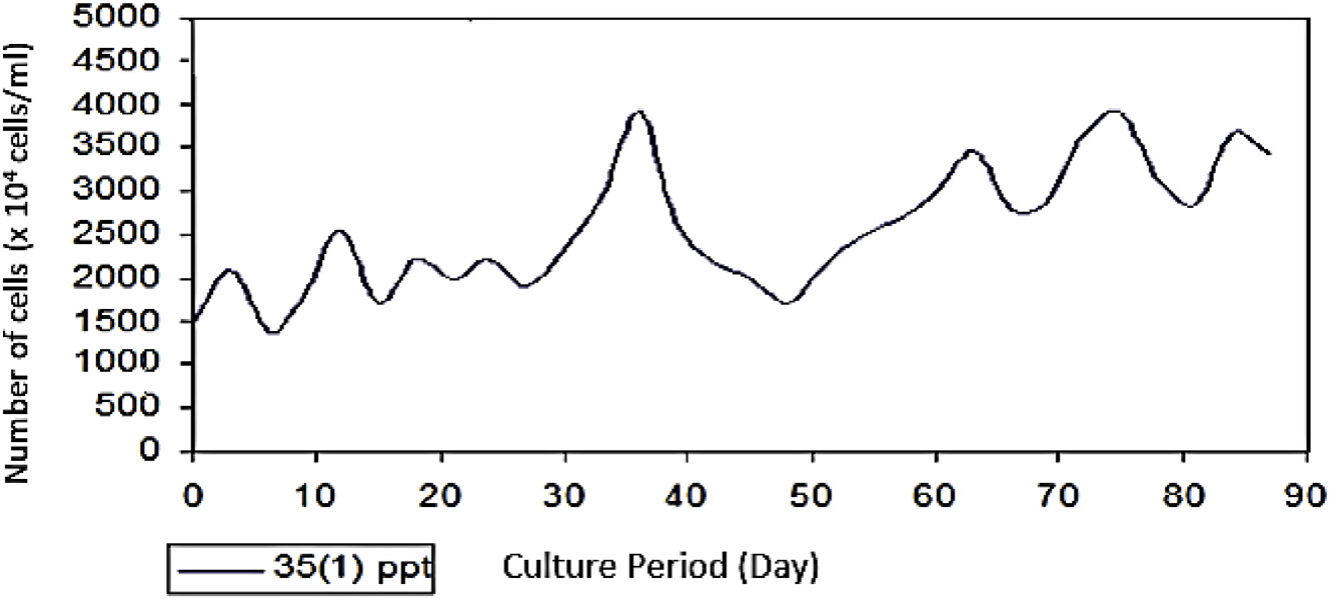
*Prochloron* cells growth at salinity of 35 ppt.

**Fig. 9:**
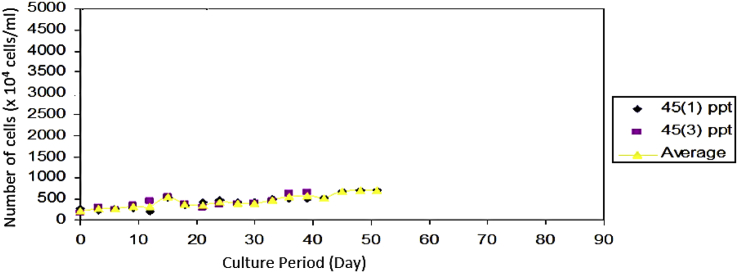
*Prochloron* cells growth at salinity of 45 ppt.

As seen on Fig. 6, *Prochloron* cultured in the medium with a salinity of 25 ppt had a population growth rate of 52.44 × 10^4^ cells/ml. Fig. 7 that shows that *Prochloron* cultured in media with a salinity of 30 ppt had a population growth rate of 45.33 × 10^4^ cells/ml. As seen in Fig. 8, *Prochloron* cultured in the medium with salinity of 35 ppt had a population growth rate of 52.44 × 10^4^ cells/ml. Fig. 9 shows that *Prochloron* cultured in medium with salinity of 35 ppt had a population growth rate of 61,66 × 10^4^ cells/ml.

**Fig. 10:**
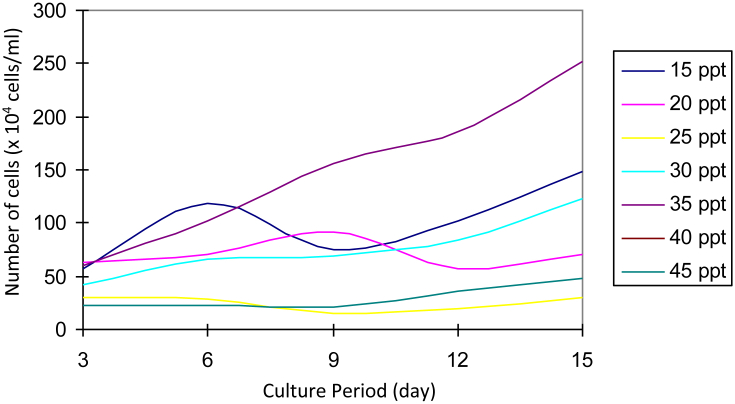
Growth of *Prochloron* cells after being given fresh media on day 90.

After being cultured for 3 months, refreshment was carried out by adding 10 ml of fresh medium for each salinity. The addition of the media was done so that the *Prochloron* cells that had been successfully purified could later be used as stock in the laboratory. Based on observation made on day 3 to day 15 (Fig. 10), it was observed that after the addition of fresh medium, the cells grown at 20 ppt salinity experienced stress because the cell density reduced. However, cells grown at 15 and 35 ppt salinity grew well.

## 2 Experimental design, materials, and methods

### 2.1 The source of *Prochloron* cells stock

*Prochloron* cells were obtained from stocks maintained at the Molecular Biology and Marine Pharmaceutics Laboratory, Faculty of Fisheries and Marine Sciences, Sam Ratulangi University. These *Prochloron* cells were initially isolated from Ascidian originating from the Malalayang coastal waters. The cells were initially cultured in the Hirata medium at 25 °C, 20 ppt salinity under continuous lighting with 20 W fluorescent lamp.

### 2.2 Culture medium

Medium used in this experiment was natural sea water enriched with Hirata medium [4] with the composition listed in Table 1. The procedure for preparing the media is described as follows: the three medium reagents were diluted beforehand in 100 ml aquadest. Two milliliters of each reagent were added into a series of salinity levels (15, 20, 25, 30, 35, 40, and 45 ppt) which were prepared by diluting sterile natural seawater with distilled water. In the preparation of solid media, 4 gr of agar was added into each medium with different salinity. The media were autoclaved at 121 °C for 15 minutes. Each agar medium was poured into Petri dish with the thickness of ± 3–5 mm and allowed to cool and solidify.

**Table 1:** Composition of Hirata medium (Hirata, 1974).

No.	Reagents	Concentration (ppm)
1.	NH_2_SO_4_	122.6
2.	Na_2_HPO_4_.12H_2_O	23
3.	Clewat 32	15
	FeCl_3_6H_2_ (Fe)	
	MnCl_2_4H_2_O (Mn)	
	CuSO_4_5H_2_O (Cu)	
	(NH_4_)_6_Mo_7_O_14_4H_2_O (Mo)	
	H_3_Bo_3_ (Bo)	
	CoCl_2_6H_2_O (Co)	
	EDTA	

### 2.3 Isolation of pure single cells of *Procholoron*

The purification method used was the dilution and the picking up method [5]. One drop of *Prochloron* suspension stock was put into each of 7 tubes containing 5 ml enriched sea water with different salinity. The cells that successfully grew were observed under a microscope. If it was in accordance with the desired characteristics of *Prochloron*, the cells were immediately transferred onto the solid media by spreading evenly 3 drops of the medium containing *Prochloron* using L-glass. The media were incubated in a culture cabinet equipped with TL 20 Watt lighting at 25 °C.

The next step was transferring the cells from solid media into liquid media. Colonies that successfully grew on solid media (green colonies) were taken using sterile inoculation needles and then put into 3 test tubes containing 5 ml of sea water media with different salinity enriched with Hirata medium. *Prochloron* cells in each of the test tubes were obtained from different Petri dishes. *Prochloron* cell growth was observed every 3 days by counting the number of cells using a hemacytometer under microscope. Each cell in the square box in the middle consisting of 400 small squares was calculated. If the amount of *Prochloron* is N, the density was N x 10^4^ (cell/ml). To facilitate the calculation, a hand counter was used.

### 2.4 Data analysis

The results obtained are presented in the form of tables and graphs of the relative growth rate of the population of each salinity calculated using the Effendie formula (1979) as follows:

$$\text{SR}=\frac{\text{Nt}-\text{No}}{\text{Nto}}\times 100\%$$

where

SR = relative population growth rate.

Nt = population at t day.

N_o_ = population at 0 day.
